# Who moderate the relationship between executive functions and quality of life among adults with and without adhd: structural equation model

**DOI:** 10.1192/j.eurpsy.2024.784

**Published:** 2024-08-27

**Authors:** N. Grinblat, S. Rosenblum

**Affiliations:** ^1^Department of Occupational Therapy, Faculty of Social Welfare & Health Sciences, University of Haifa, Haifa, Israel

## Abstract

**Introduction:**

Literature evidences indicates that adults with attention-deficit/hyperactivity disorder (ADHD) are struggling with executive functions deficiencies, organization in time deficits, low sleep quality, and poor quality of life (QoL). However, it is not clear how those factors associate and interact with each other.

**Objectives:**

This study aims to compare those factors as well as the relationships between them, among adults with and without ADHD using structural equations modeling (SEM).

**Methods:**

Sixty-nine adults with ADHD and 52 matched controls (ages 20-46) completed the Behavior Rating Inventory of Executive Function-adult version (for executive functions), Time Organisation and Participation Scale (for organization-in-time), Mini Sleep Questionnaire (for sleep quality), and Adult ADHD Quality of Life questionnaire (for QoL).

**Results:**

Compared to adults without ADHD, adults with ADHD showed significantly poorer executive functions, organization-in-time, sleep quality and QoL. The SEM indicated that sleep quality and organization-in-time domains mediated the relationship between executive functions abilities and QoL. This SEM explained 79% of the QoL variance for adults with and without ADHD.

**Conclusions:**

Understanding the role of organization-in-time and sleep quality as mediators between executive functions and quality of life emphasize the unique challenges of adults with ADHD, which deals with deficiencies at those factors. Those findings call for including these factors in evaluation and intervention processes to improve QoL and this population’s global health.

**Disclosure of Interest:**

None Declared

